# Supplementary figures and images for: Tamoxifen Sensitizes Acute Lymphoblastic Leukemia Cells to Cannabidiol by Targeting Cyclophilin-D and Altering Mitochondrial Ca2+ Homeostasis
Source: Int J Mol Sci. 2021 Aug 13;22(16):8688. doi: 10.3390/ijms22168688 (PMC8395529; doi:10.3390/ijms22168688)

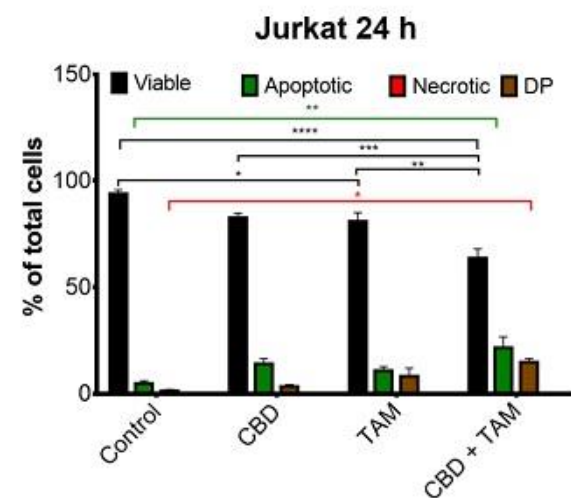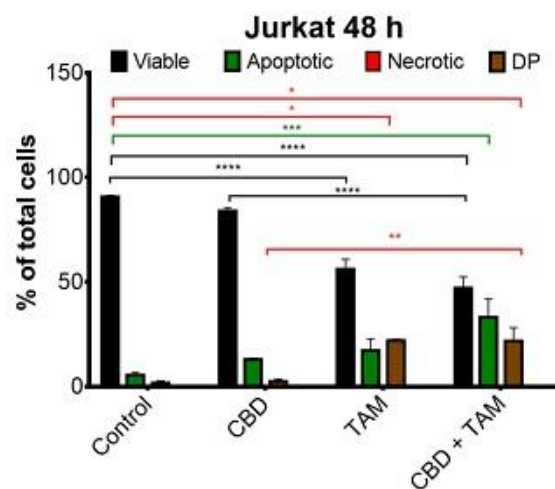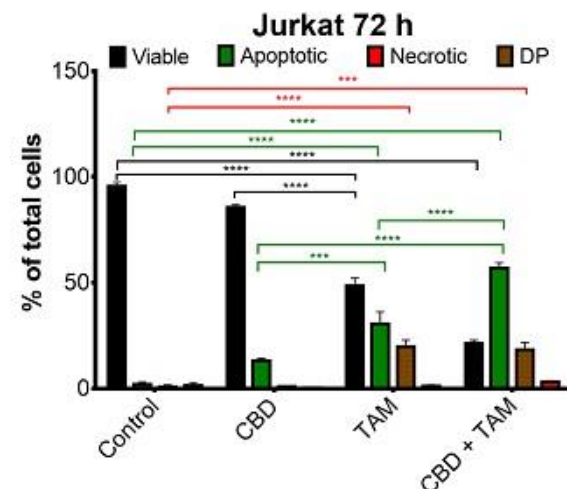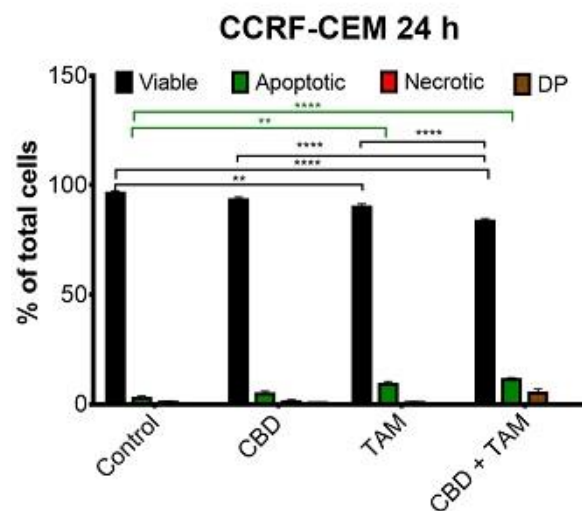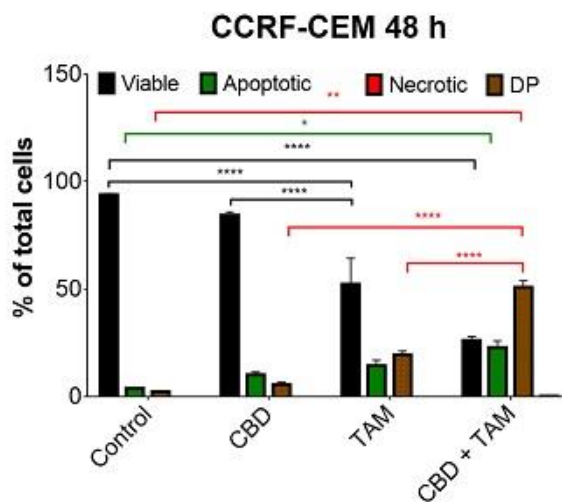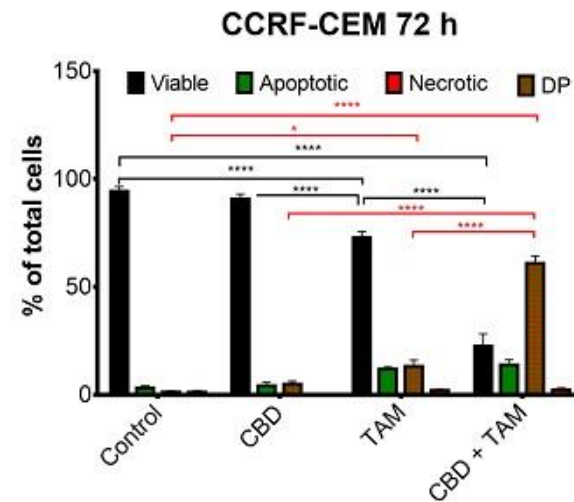

Supplement: Supplementary file 1 [file ijms-22-08688-s001.zip › ijms-1302062-supplementary.pdf]
